# The effect of self-focus on personal and social foraging behaviour

**DOI:** 10.1093/scan/nsy057

**Published:** 2018-08-23

**Authors:** George Zacharopoulos, Amitai Shenhav, Sara Constantino, Gregory R Maio, David E J Linden

**Affiliations:** 1 Cardiff University Brain Research Imaging Centre, School of Psychology, Cardiff University, Cardiff, United Kingdom; 2 Department of Cognitive, Linguistic and Psychological Sciences, Brown Institute for Brain Science, Brown University, Rhode Island, United States; 3 Department of Psychology, New York University, New York, United States; 4 Neuroscience and Mental Health Research Institute, Cardiff University, United Kingdom; 5 MRC Centre for Neuropsychiatric Genetics and Genomics, Cardiff University, United Kingdom; 6 Department of Psychology, University of Bath, Bath, United Kingdom; 7 Department of Experimental Psychology, University of Oxford, Oxford, United Kingdom

**Keywords:** foraging, economic decision-making, self-focus, individual differences, fMRI

## Abstract

The continuous balancing of the risks and benefits of exploiting known options or exploring new opportunities is essential to human life. We forage for new opportunities when they are deemed to be more attractive than the available option, but this decision to forage also entails costs. People differ in their propensity to exploit or forage, and both the social circumstances and our individual value orientations are likely influences. Here, participants made foraging decisions for themselves and for a charity of their choice in two paradigms: one that features two distinct modes of decision-making (foraging *vs* classical economic decision-making) and one which is more directly related to the classical animal foraging and ethology literature. Across both paradigms, individuals who possessed a stronger self-focused value orientation obtained more rewards when they were allowed to forage for themselves rather than the charity. Neuroimaging during the tasks revealed that this effect was associated with activity in the dorsal anterior cingulate cortex (dACC) in that more self-focused individuals showed lower activity in dACC for the self-condition relative to the other condition. This evidence reveals a dynamic interplay between foraging outcomes and the higher-order value system of individuals.

## Introduction

Many life decisions can be conceptualised as foraging problems (Charnov, 1976; Constantino and Daw, 2015) where the choice is between the exploitation of certain options and the exploration of less certain ones. Employment decisions, mate selection and internet searches are just a few scenarios wherein people must choose whether to engage with the currently available options or to search for alternative ones. To solve this type of problem, an ideal forager compares the value of two strategies—engaging with the currently available option or foregoing it to search for alternatives—and chooses the one with the highest value. The optimal solution (Marginal Value Theorem, Charnov, 1976) to the foraging problem requires comparing the value of the current option to the overall value of the alternative, foraging environment.

However, maximising personal gain is not the only determinant for value decisions. Humans and other social animals often forage on behalf of conspecifics. It is thus likely that foraging decisions will be influenced by an individual’s social learning history and social disposition. We present the first study to contrast personal and social foraging in order to examine their potential behavioural and neural differences. How much we value another’s welfare may crucially influence foraging decisions within the human social environment, but research has not studied whether foraging for others involves substantively different processes from foraging for oneself.

Outside of the foraging context, several studies have investigated behavioural differences between self *vs* other scenarios. In the context of moral decision-making, Crockett *et al.* (2014) contrasted how much money people will sacrifice to reduce the number of painful electric shocks delivered to either themselves or to an anonymous stranger. Results indicated that people sacrifice more money to reduce a stranger’s pain than their own. Mengarelli *et al.* (2014) contrasted monetary risk preferences involving individuals’ own money or another person’s money and found reduced loss aversion bias when the choices involved another person’s money. In the context of perception and attention (see Sui and Gu, 2017, for a review), when participants were asked to make a classification of faces as self, friend or stranger, Keyes (2012) found that classification of self-faces was faster than classifying faces of other people. Moreover, an enhanced attention marker (N1) and a reduced decision-making marker (P3) was observed for own compared to others’ faces in a study examining the effects of facial cues on the orienting of visual attention with event-related potentials (Liu *et al.*, 2015).

Moreover, a number of studies have investigated self *vs* other differences using neuroimaging. In particular, several studies have examined the neural basis of charitable decision-making. Moll *et al.* (2006) showed that donating money to a charity recruited the mesolimbic reward system in a similar way as when monetary rewards were obtained. Izuma *et al.* (2009) found high ventral striatal activations when individuals made charitable donations in public. Tusche *et al.* (2016) identified dissociable roles of anterior insula and temporoparietal junction for affective empathy and cognitive perspective taking, respectively, while relating these routes to intraindividual and interindividual differences in altruistic behaviour.

Regions across the anterior cingulate cortex (ACC) have recently moved into the focus of studies comparing the processing of reward for self *vs* other as well as foraging processing in general. However, ACC consists of several subregions and previous research showed that there is more than one signal coming from ACC, and that some of the signals may arise from these different subregions. Using computational modelling and functional magnetic resonance (fMRI) in a reward learning task, Lockwood *et al.* (2016) showed that the subgenual anterior cingulate cortex (sgACC) was associated with reward learning only when individuals acted in a prosocial context, and this region signals a prosocial prediction error. Moreover, individuals who scored higher in trait empathy were faster in learning to benefit others, and they exhibited a sgACC response that was more selective for prosocial learning. Consistent with the growing focus on the ACC, Apps *et al.* (2016) presented a model based on vicarious motivation and error processing whereby a specific region of ACC gyrus (ACCg) is involved in costs, benefits and errors during social interactions. Finally, ACC was also involved in a neuroeconomics study that investigated the self and other processing in the context of the ‘trust game’ (King-Casas *et al.*, 2005). When participants made investment decisions as the investor (self-phase) and when they played the trustee role and observed the investors’ decisions (other-phase), there was elevated activation in mid-cingulate cortex (MCC) and ACC, respectively. In addition, dorsal regions of ACC have been implicated in foraging contexts in both humans (Kolling *et al.*, 2012) and primates (Hayden *et al.*^2011^), though the nature of this role (i.e. the computations that take place during foraging) remains under debate (Shenhav *et al.*, 2014, 2016a, 2016b; Kolling *et al.*, 2016). Given this wide body of work, we predict that ACC should be involved in personal and social foraging processing.

**Fig. 1:**
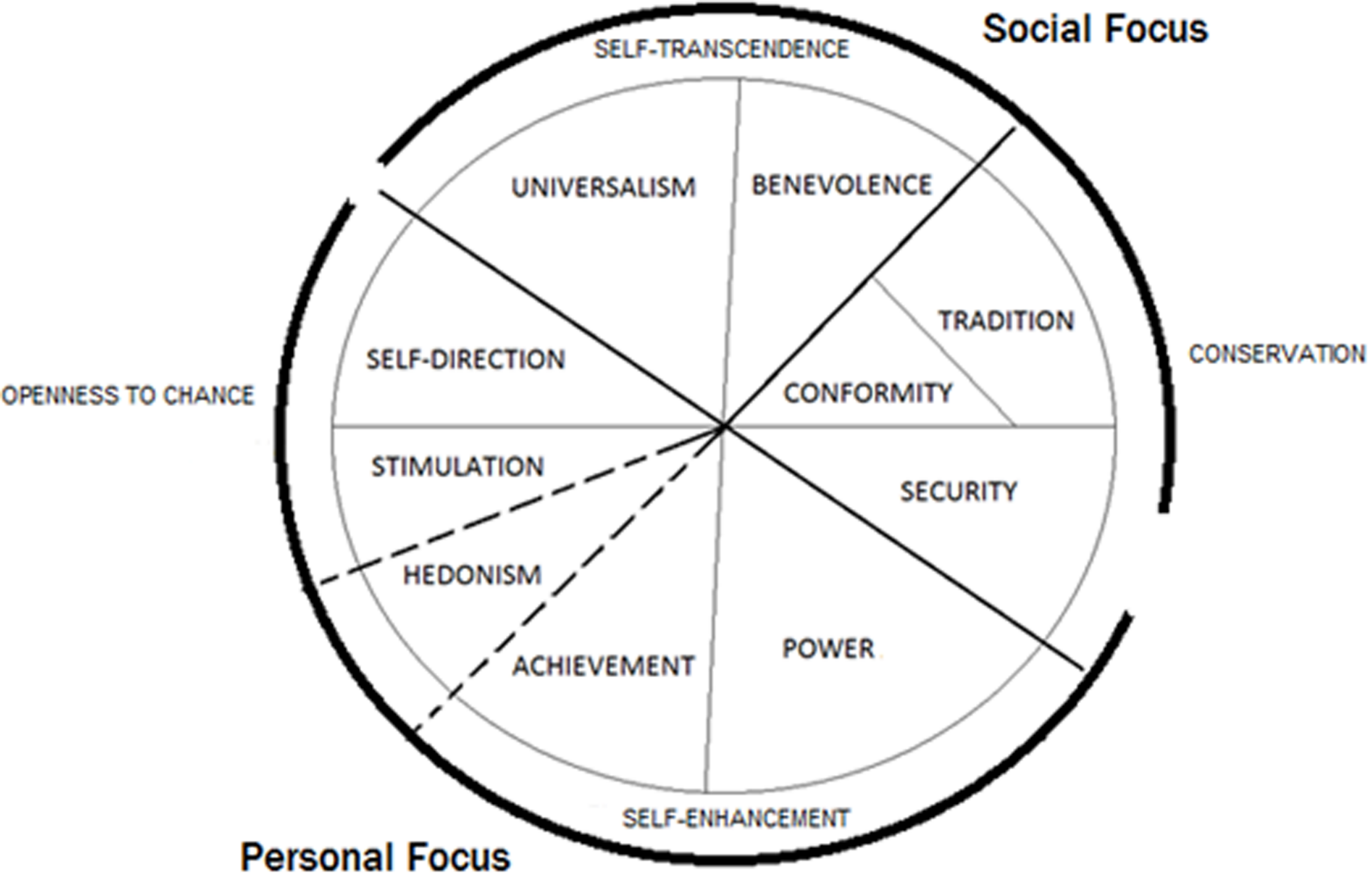
The circumplex structure of human values (modified from Schwartz, 1992). Self-focused individuals score high on the value types self-direction, stimulation, hedonism, achievement, power and security, while the social-focused individuals score high on universalism, benevolence, tradition, conformity and security.

To understand the aforementioned distinction between social and personal foraging behaviour, it is imperative to also consider the role of individual differences. Personal value orientation is an individual difference variable that possesses a particularly close conceptual connection to this self–other distinction. Value orientations are defined as ‘the principles or standards of behaviour, one’s judgement of what is important in life’ (Soanes and Stevenson, 2003). These orientations are frequently measured utilising Schwartz’s (1992, Schwartz *et al.*, 2012) Circumplex Model of Values, which has been validated in over 80 nations and has been subjected to diverse experimental, longitudinal and cross-sectional tests (Maio, 2010). The model posits the existence of 10 types of human values (Figure 1), with each expressing specific motives organised according to their motivational conflicts and compatibilities. Of particular relevance for our interests is the contrast between values with a personal *vs* a social focus (Schwartz *et al.*, 2012). The personal end of the dimension features values promoting power, achievement, stimulation, hedonism, self-direction and security. In contrast, the social end features values promoting benevolence, universalism, tradition, conformity and security. Of particular relevance here is the contrast between self-focused values and social-focused values in this model (Schwartz *et al.*, 2012). The self-focus dimension comprises the egocentric human values of self-direction, stimulation, hedonism, achievement, power and security. Conversely, the social-focus dimension incorporates a high regard for social norms (tradition, conformity, security) and well-being of others (benevolence, universalism).

Individuals with a personal focus are more concerned with outcomes for self, whereas those with a social focus are concerned with outcomes for others or for established institutions (Schwartz *et al.*, 2012). To this end, this personal *vs* social distinction in values should be pivotal in how people construe personal (foraging for oneself, see below) *vs* social (foraging on behalf of others) foraging tasks. While personal foraging should be influenced by how people assign reward value to choices that have varying degrees of personal costs and benefits, social foraging behaviour (foraging for others) should be influenced by how people assign reward value to choices that have varying degrees of costs and benefits for others. Previous studies have demonstrated individual variation in foraging behaviour for the self (Kolling *et al.*, 2012; Shenhav *et al.*, 2014; Constantino and Daw, 2015), but have provided little information on motivational determinants of this variation. Based on previous findings in prosociality and classical economic decision-making (Brosch *et al.*, 2011; Lockwood *et al.*, 2017), we would expect self-focused individuals to attempt to gather more money for themselves (personal foraging) than for charity (social foraging). However, it is less clear whether a self-focused individual would actually obtain more reward during personal foraging than a social-focused person.

We thus designed two experiments that would enable us to address these issues. In both experiments, participants alternated between foraging for themselves and foraging for a charity of their choice. In Experiment 1, participants decided whether to engage with a given choice of options or to keep looking for better options (foraging). When they chose to engage with a given option, they were asked to make a classical economic decision between two outcomes with known reward magnitudes and probabilities. In Experiment 2, participants were presented with a video simulation of apple harvesting. They were shown an image of an apple tree and had to decide whether to harvest it for apples and incur a short harvest delay, or move to a new tree and incur a longer travel delay. The design of Experiment 1 crucially involved two distinct modes of decision-making, and thus allowed us to address the influence of human values on foraging and classical economic decision-making separately. The design of Experiment 2, with its traditional patch depletion environment, includes only the foraging stage (equivalent to Stage 1 of Experiment 1, which takes cross-sections through the traditional patch-depletion environment on each trial). This design was used to test whether similar patterns of behaviour are obtained in a foraging design directly related to the classical animal foraging literature (e.g. Charnov, 1976; Stephens and Krebs, 1986). At the behavioural level, we predict in both Exp 1 and Exp 2 that self-focused individuals should attempt to gather more money for themselves (personal foraging) than for charity (social foraging). Unlike Exp 2, Exp 1 was an fMRI study. With respect to the imaging hypotheses, as noted before given the relevance of ACC in the self *vs* other processing together with evidence of foraging computations within the ACC in both humans (Kolling *et al.*, 2012) and primates (Hayde *et al.*, 2011), we predict that ACC should be involved in personal and social foraging processing.

## Materials and methods

### Participants

Thirty undergraduate and post-graduate university students between 18 and 37 years of age (9 males) took part in the study. Participants were informed that the study investigates the neural (Experiment 1) and behavioural (Experiments 1 and 2, instructions of which can be seen in Supplementary Information 1) mechanisms of foraging behaviour. All participants completed Experiment 1 before Experiment 2. For the behavioural analysis in Experiment 1, three participants were excluded because of incomplete scanning sessions, and one participant was excluded because she foraged fewer than seven times (i.e. only once) during social foraging (7, exclusion criterion). Seven additional participants were excluded from the neuroimaging analysis in Experiment 1 because of excessive motion; that is, at least a single image exceeded 2 mm during realignment within a single run. In Experiment 2, one participant was excluded because of excessive time outs (i.e. more than three standard deviations above the mean). Overall, 25 participants were included for the behavioural analysis across both experiments, and 18 subjects were included for the fMRI analysis in Experiment 1. The study was approved by the Cardiff University School of Psychology ethics committee, and all participants gave written informed consent.

### Experimental design

#### Charity selection

Participants were informed during the instruction phase (for full instructions, see Supplementary Information 2) that the number of points (Experiment 1) or apples (Experiment 2) would be converted into real money at the end of the experiment and that the reward obtained during personal foraging would be paid to them (on top of the fixed participation payment of £15 in Experiment 1 and £6 in Experiment 2), while the reward obtained during social foraging would be given to the charity. Participants were then asked to select the charity of their choice from a list including the following charities: British Red Cross, Save the Children Fund, Oxfam, The Salvation Army, Cancer Research UK and Macmillan Cancer Support. Participants were free to choose different charities in the two experiments. The behavioural data in Experiment 1, but not those in Experiment 2, were obtained inside the fMRI scanner.

#### Experiment 1

**Fig. 2:**
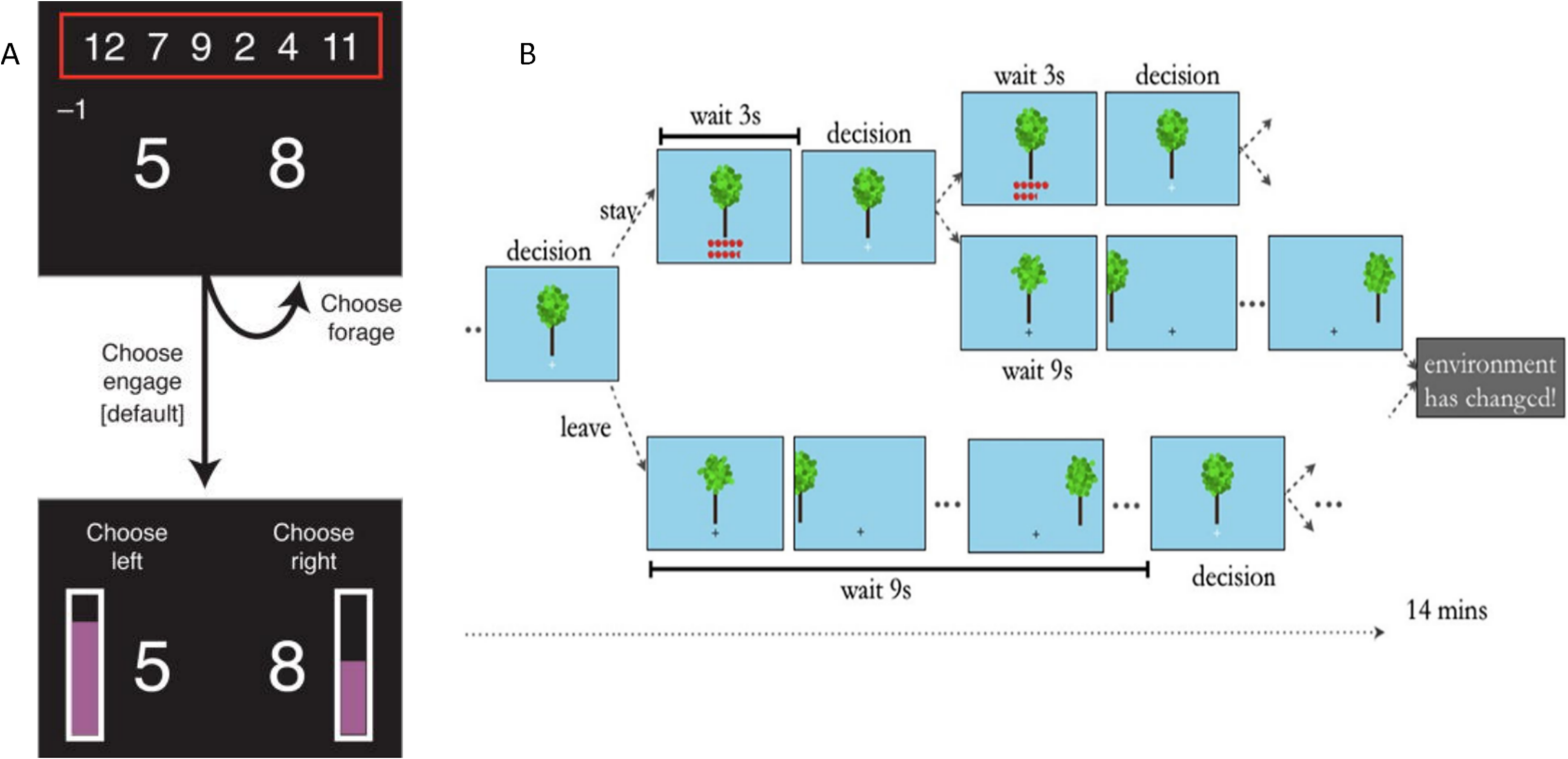
Graphical depictions of a trial in Experiment 1 (Panel A, adapted from Shenhav *et al.*, 2014) and Experiment 2 (Panel B, adapted from Constantino and Daw, 2015).

In Experiment 1, participants performed a two-stage decision-making task (Kolling *et al.*, 2012; Shenhav *et al.*, 2014, Figure 2, Panel A). In Stage 1, participants decided whether to engage with a given choice of options or to keep looking for better options (foraging). In Stage 2, participants made a classical economic decision between two outcomes with known reward magnitudes and probabilities. On each trial in Stage 1 (upper panels, Figure 2A), participants were offered a pair of potential rewards (large numbers). They could choose to forage for a better pair of rewards from the set shown at the top of the screen (smaller numbers in the red box), in which case a random pair from that set was swapped with the current offer. Participants who made this choice would incur a forage cost (shown on the left, below the red box) and a delay until the new choice was shown. Participants could forage any number of times (or not at all) before opting to proceed to Stage 2 (lower panel). After they entered Stage 2, a probability was randomly assigned to each of the reward options (height of violet bar beside each number) and participants were prompted to choose one of the magnitude-probability pairs. Participants received the outcome of the gamble on each Stage 2 trial, and these were displayed as accumulating points at the bottom of the screen (not shown).

Participants completed a total of eight fMRI runs (four personal foraging, four social foraging). Before each of the eight blocks, a short message on the screen told participants whether the forthcoming block was personal or social. Participants experienced the foraging conditions in one of two orders: (1) personal, social, personal, social, social, personal, personal, social or (2) social, personal, social, personal, personal, social, social, personal. A block typically lasted for 11 min.

The analysis of the behavioural data of Experiment 1 focused on the reward during (1) personal foraging (i.e. the total number of points obtained for self during the four personal foraging runs), (2) social foraging (i.e. the total number of points given to the charity during the four social foraging runs) and (3) the difference of the two (i.e. the reward obtained for self minus the reward given to the charity).

#### Experiment 2

In Experiment 2, participants completed an adapted version of a virtual patch-foraging task (Figure 2B; 2). Each run consisted of approximately 50 trials. Participants foraged (Figure 2B) for apples in four 14-min (i.e. 7 m Personal Foraging, 7 m Social Foraging) virtual patch-foraging orchards (i.e. Long-Shallow, Long-Steep, Short-Shallow, Short-Steep). The recipient of reward (i.e. self, charity) was signalled with a letter (‘S’, self, or ‘O’, other) that was presented throughout each block (not shown in the figure).

On each trial, participants were presented with a tree and had to decide whether to harvest it for apples and incur a short harvest delay, or move to a new tree and incur a longer travel delay. Harvests at a tree earned apples, albeit at an exponentially decelerating rate. Similar to Constantino and Daw’s study (2), we varied the quality of the foraging context by manipulating two environmental parameters: depletion rate and travel time. The depletion rate determines the rate at which earned apples decrease with subsequent harvest decisions at a given tree. It is a fixed multiplicative decay κ, such that if a participant harvests eight apples in the current trial, the number of apples to be harvested in the next trial will be the depletion rate multiplied by 8. By manipulating the depletion rate, we created one environment with fast depletion (steep) and one with slower depletion (shallow). Additionally, we created two more types of orchards—long (9 s) and short (6 s)— by manipulating the travel time, the time it takes to travel to a new tree. Combining these two manipulations resulted in the four orchard-types that participants visited during the task: Long-Shallow, Long-Steep, Short-Shallow and Short-Steep. Apart from the depletion rate and travel time, all of the other environment parameters remained the same across orchards (Supplementary Information 2).

New trees were drawn from a Gaussian distribution and the environmental richness or opportunity cost of time was varied across blocks by changing the travel time and/or the apple depletion rate (see below). The quality of the tree, depletion rate and richness of the environment were *a priori* unknown to the subject. The aim of the participants was to maximize their reward (i.e. number of apples) for themselves or for a charity of their choice, depending on their experimental condition. Similar to Experiment 1, this was a within-subject design.

Participants foraged in each orchard for 14 min (7 min personal and 7 min for social foraging). Similar to Experiment 1, Experiment 2 focused on the reward during (1) personal foraging (i.e. the total number of apples obtained for self during the personal foraging), (2) social foraging (i.e. the total number of apples given to the charity during the social foraging) and (3) the difference of the two (i.e. the reward obtained for self minus the reward given to the charity). In addition to the reward, we looked at individual exit thresholds, defined as the mean number of apples at which the participants choose to switch to a new tree in each of the environment types. This was calculated by averaging the number of apples at exit across trees in a given orchard. For example, an exit threshold of 9 means that participants tended to leave a tree when the last harvest yielded 9 apples. Each orchard has an optimal, average reward-maximizing exit threshold given by the Marginal Value Theorem (Charnov, 1976). A higher than optimal empirical exit threshold signals underharvesting bias (i.e. leaving trees too early), while one that falls below the optimal threshold signals an overharvesting bias (i.e. staying with trees too long). For the purposes of this study, we calculated the exit threshold separately for personal and social foraging.

### MRI data acquisition

All MRI Data were acquired at the Cardiff University Brain Research Imaging Centre on a 3 T GE SignaHDx system (General Electric, Milwaukee, USA) equipped with an 8HR Brain parallel head coil for radio frequency transmission/reception.

#### Structural MRI

Anatomical high-resolution T1-weighted volume scans (1 mm^3^) were acquired using a fast spoiled gradient echo (FSPGR) 3-D sequence (repetition time (TR) = 7.849 ms; echo time (TE) = 2.984 ms; field of view = 256 × 256 mm; voxel size = 1 × 1 × 1 mm).

#### Functional MRI

Functional images were acquired with a gradient-echo EPI sequence (TR 3000 ms, TE = 30 ms, flip angle 87°, gap = 1 mm, number of slices = 43, voxel dimension = 3.5 × 3.5 × 4.4, tilted 15° relative to the AC/PC plane).

### MRI data pre-processing

Imaging data were analyzed in SPM8 (Wellcome Department of Imaging Neuroscience, Institute of Neurology, London, UK). Functional volumes were motion corrected, normalized to a standardized (MNI) template (including resampling to 2-mm isotropic voxels), spatially smoothed with a Gaussian kernel (8-mm FWHM) and high-pass filtered (0.01-Hz cut-off). Separate regressors were included for the Stage 1 and Stage 2 decision phases. These regressors were all modelled as stick functions (which sets the duration of events to 0 and the event is modelled using a canonical hemodynamic response function). The main analyses probed the difference between personal and social (i.e. contrast self minus charity conditions) in two independent analysis one during foraging (Stage 1) and the other during classical economic decision-making (Stage 2). The GLM#1 featured the two main predictors (Stage 1, foraging and Stage 2, classical economic decision-making) but additionally included separate parametric regressors. Stage 1 featured three parametric regressors: (i) task difficulty (defined as the negative absolute value of the log-odds of choosing to forage *vs* engage on each trial based on the choice values), (ii) search evidence and (iii) search cost (i.e. the amount of points the participant will deterministically lose if they choose to forage), while Stage 2 featured one parametric regressor, relative value (i.e. the difference between reward magnitude of the left option * reward probability of the left option and the reward magnitude of right option * reward probability of the right option). The parametric modulators were standardized (i.e. *z*-scored) at the subject level before they were entered into the General Linear Model (GLM) and we disabled Statistical Parametric Mapping (SPM’s) default orthogonalization option which sequentially orthogonalizes the regressors in a hierarchical fashion during the first-level analysis. All analysis presented below refer to GLM#1 apart from the Supplementary Information 6 (where we used GLM#2 which was identical to GLM#1 but did not feature any parametric modulators). The cluster-level results were obtained using both SPM’s random field theory and the toolbox Statistical Non-Parametric Mapping (SnPM, see Supplementary Information 3) within SPM8. SnPM uses the GLM to construct pseudo *t*-statistic images, which are then assessed for significance using a standard non-parametric multiple comparisons procedure based on randomisation/permutation testing. All results presented below involve clusters that survived Family Wise Error (FWE)-cluster level correction at an initial uncorrected voxel-wise *P*-value cluster forming threshold of 0.001. Our approach is consistent with current guidelines on the reporting of whole-brain MRI data (Roiser *et al.*, 2016).

### Human values

Participants completed the Schwartz value survey (SVS; Schwartz, 1992), a 56-item scale that can be used to measure the value types shown in Supplementary Information 1. Participants were asked to rate the importance of each of the 56 values as a guiding principle in their lives, using a quasi-bipolar 9-point scale ranging from −1 (opposed to my values), 0 (not important), 4 (important) to 7 (of supreme importance). Examples of SVS items are as follows: ‘Equality: Equal opportunity for all’ (Universalism); ‘Pleasure: Gratification of desires’ (Hedonism); ‘Obedient: Dutiful meeting obligations’ (Conformity). The average score across the 56 items was calculated and subtracted from each of the 56 initial raw scores, prior to calculating the average of the value scores within each of the 10 value types. Schwartz recommends this procedure to help control for superfluous individual variations in rating styles (Schwartz, 1992). To create the self-focus score, we calculated the average score on self-direction, stimulation, hedonism, achievement, power and security values. To calculate the social-focus score and we calculated the average score of universalism, benevolence, tradition, conformity and security values.

## Results

### Behavioural parameters

We first tested whether we replicated the behavioural findings of the original studies (Shenhav *et al.*, 2014; Constantino and Daw, 2015). In Exp 1 we replicated the overall tendency (Kolling *et al.*^2012^, Shenhav *et al.*, 2014) of individuals to show an engage *vs* forage bias during Stage 1, which was consistent in both the personal (*P* < 0.05) and social (*P* < 0.05) foraging. Similarly, we replicated the main exit threshold findings in Exp 2. Namely, in the short *vs* long travel time orchards participants showed a higher exit threshold (*P* < 0.05). Similarly, in the shallow *vs* steep orchards, participants showed a higher exit threshold (*P* < 0.05). Importantly, these patterns were also replicated significantly in the self and other conditions separately. Having thus replicated the behavioural effects of the original studies, we proceeded to investigate any behavioural differences between the personal and social foraging conditions.

We then tested for any differences in specific behavioural parameters in the paradigms, such as the overall reward earned, number of foraging instances, exit threshold and reaction times between personal *vs* social conditions. In Exp 1, we looked at the following: (i) the Stage 1 intercept (the constant from the foraging binary logistic regression predicting foraging *vs* engaging based on six continuous predictors the beta-weights of which are plotted here, the higher engaging value, the lower engaging value, highest foraging value, average foraging value, lowest foraging value, search cost); (ii) the Stage 2 intercept (the constant from the classical economic decision-making; the binary logistic regression predicted the left or right option, with beta-weights for reward and probability); (iii) the number of overall points; (iv) overall cost incurred; (v) number of overall forages; and (vi) reaction times. In Exp 2, we looked at the following: (i) the number of overall points; (ii) overall cost incurred; (iii) number of overall forages; (iv) exit thresholds; and (v) reaction times. When contrasting self *vs* other conditions, none of these behavioural parameters was significant.

**Figure 3:**
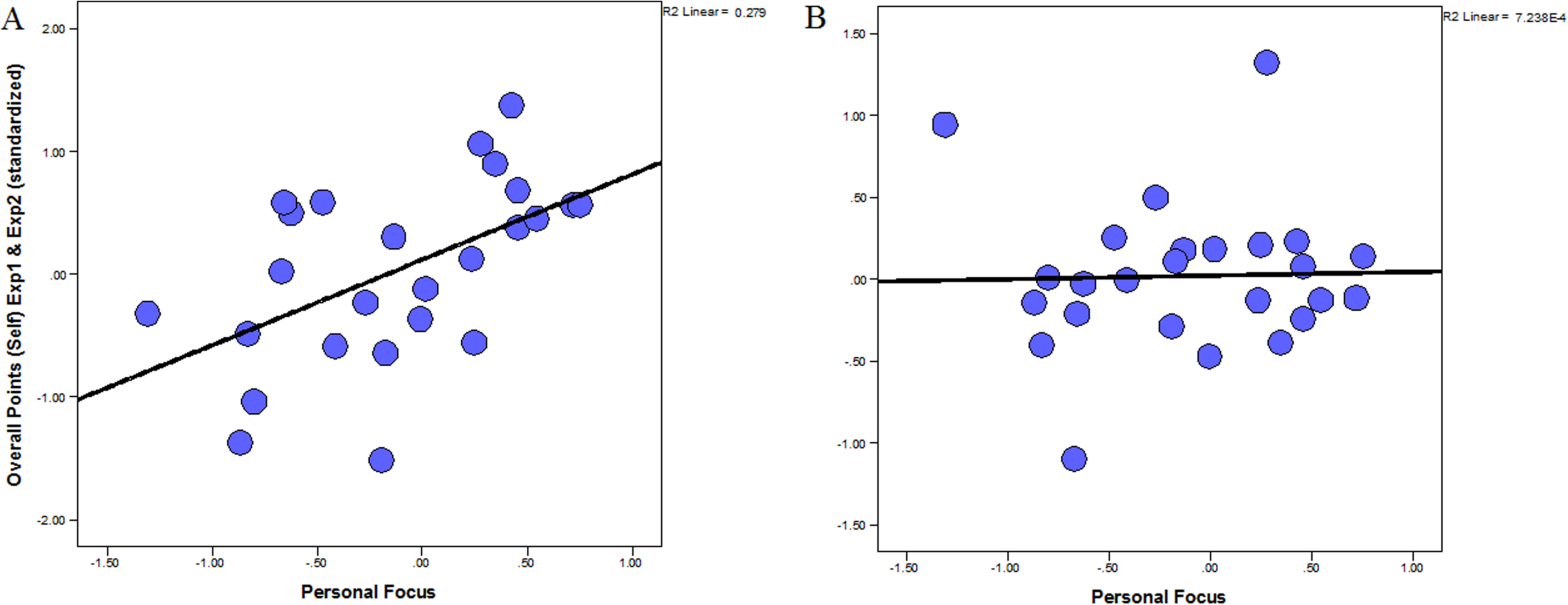
Scatterplots depicting the associations between human-value orientation and overall reward obtained during personal and social foraging (across Experiment 1 and Experiment 2). These depict a positive association between self-focused values and overall reward during personal (Panel A) but not social (Panel B) foraging for Experiment 1 and Experiment 2 combined (Panel A).

**Table 1:** Correlations between the social values and foraging indices

	SE	ST	CO	OP	Self-Focus	Social-Focus
Total Number of Points Self (Exp1)	*r* = 0.109	*r* = −0.314	*r* = −0.042	*r* = 0.442*	*r* = 0.411*	*r* = −0.249
Total Number of Points Other (Exp1)	*r* = −0.290	*r* = 0.061	*r* = −0.007	*r* = 0.001	*r* = −0.134	*r* = 0.033
Total Number of Points Self-Other (Exp1)	*r* = 297	*r* = −0.276	*r* = −0.025	*r* = 0.324	*r* = 0.402*	*r* = 0.1207
Total Number of Points Self (Exp2)	*r*_s_ = 0.383*	*r*_s_ = −0.084	*r*_s_ = −0.273	*r*_s_ = 0.330	*r*_s_ = 0.411*	*r*_s_ = 0.315
Total Number of Points Other (Exp2)	*r*_s_ = 0.141	*r*_s_ = 0.202	*r*_s_ = 0.357	*r*_s_ = 0.131	*r*_s_ = 0.164	*r*_s_ = −0.148
Total Number of Points Self-Other (Exp2)	*r* = 0.252	*r* = −0.355	*r* = 0.077	*r* = 0.156	*r* = 0.294	*r* = −0.137

### The effect of self-focus on the overall reward

We then tested whether self-focused individuals *vs* social-focused individuals obtained more reward during the personal conditions rather the social conditions. Participants who exhibited a higher self-focus value orientation indeed earned higher overall reward during personal foraging (across Experiments 1 and 2, adding the standardized overall reward in Experiment 1 and the equivalent in Experiment 2 into a single score) *r*(23) = 0.529, *P* = 0.007 (*p*_BONF_ = 0.014, given the two tests: self and social focus); Figure 3A, but not during social foraging, *r*(23) = 0.027, *P* = 0.898; Figure 3B. Indeed, these two correlations were significantly different from each other, *z*-score = 2.077, *P* = 0.038. Furthermore, the self-focus value orientation was positively associated with the difference between overall reward during personal and social foraging, *r*(23) = 0.455, *P* = 0.022.

For completeness, we conducted the same analysis separately for Experiments 1 and 2, as well as correlating these values with the second-level value dimensions that make up the self-focus and social focus: self-enhancement (SE), self-transcendence (ST), conservation (CO) and openness (OP) to change (Table 1). Self-Focus was consistently and significantly positively correlated with reward obtained for self in both Exp 1 and Exp 2.

### Searching for potential behavioural mediators of the effect of self-focus on the overall reward

After establishing a link between the self-focus score and the overall reward we investigated whether this relationship is associated with behavioural markers of OP to Experience (i.e. number of forages and the intercept of Stage 1 binary logistic regression which reflects the overall tendency of the participant towards foraging or engaging in Experiment 1 and the number of forages or exit threshold in Experiment 2) or SE (i.e. higher reaction time indicating more careful decisions), which are the two main components constituting the self-focus score. We calculated the difference in these markers (self–other conditions) and related them to the difference in the corresponding overall reward (self–other conditions), but found no significant association.

### FMRI analysis: task difficulty and effects of condition

In order to validate our neural data against previous studies (Shenhav *et al.*, 2014, 2016b), we first performed whole-brain analyses to test for correlates of foraging value and choice difficulty during the foraging stage of the task (Stage 1), collapsing across the self and charity conditions. Replicating these previous findings, we find that a region of dorsal anterior cingulate cortex (dACC) was robustly associated with the difficulty of a foraging choice (Supplementary Information 4; peak MNI coordinates: x = 6 y = 22 z = 50, *t* = 8.35; k = 640, cluster-corrected *p*FWE *=* 0.006). Having provided this initial validation, we next tested for regions that differentiated between the self and charity condition, either during foraging (Stage 1) or classical economic decision-making (Stage 2). Consistent with our behavioural null results, no brain region was differentially active for the other *vs* self condition, either in Stage 1 or Stage 2.

### FMRI analysis: effects of self-focus

**Fig. 4:**
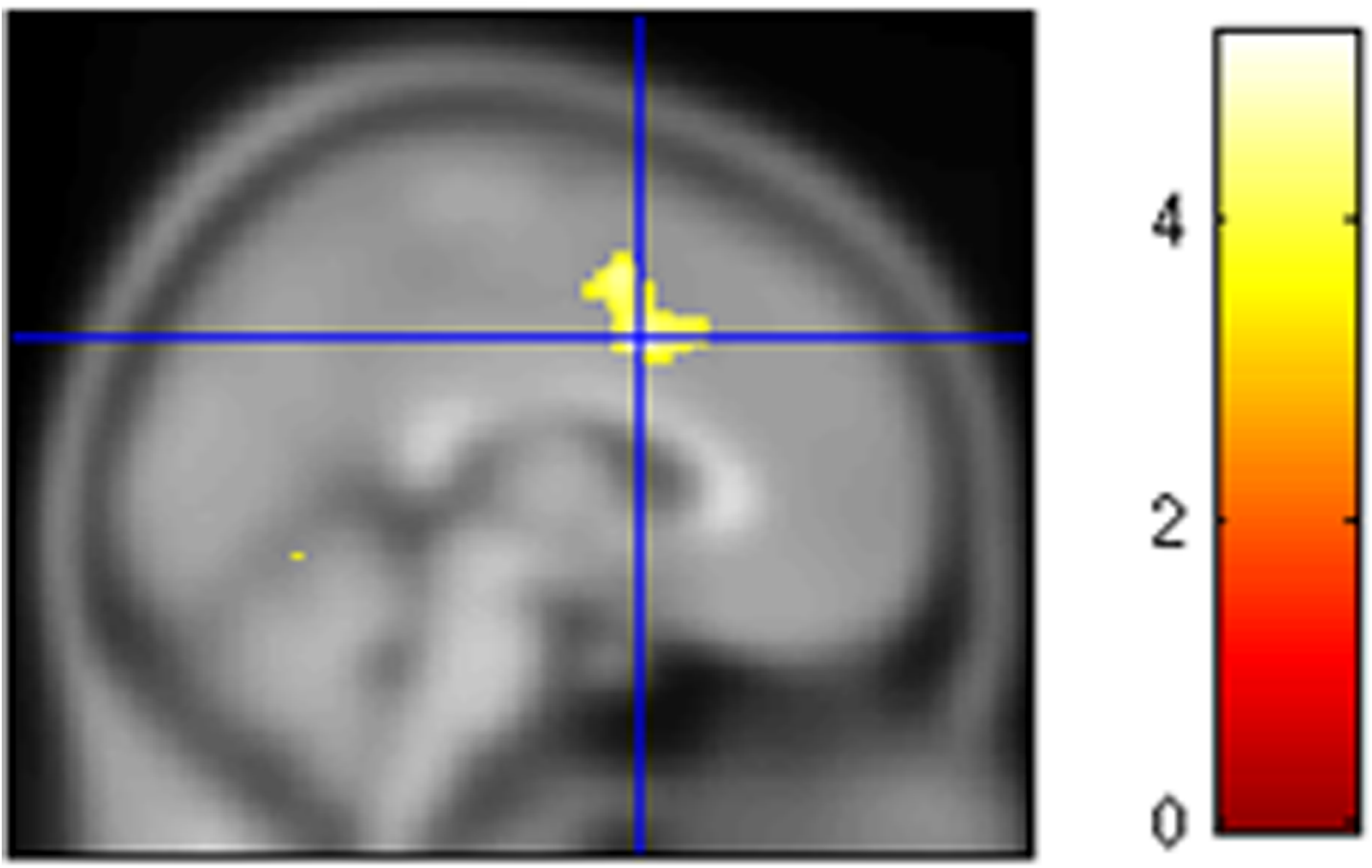
Less self-focused individuals showed greater activity in dACC for the self condition relative to the other condition.

Given our finding that the self-focus is correlated with the amount of reward obtained during the personal *vs* social conditions, we next tested whether this value orientation is associated with neural activity during the personal foraging relative to social foraging. We found that self-focus was indeed negatively related to the contrast of average neural activity during self *vs* other trials, in a cluster encompassing dACC and more central regions of the MCC (x = 2 y = 8 z = 38, k = 624; *P* = 0.011; Figure 4, (see a bar plot of the raw beta-weights and correlations between the raw beta weights and the self-focus score in the Supplementary Information 5). Individual variation in the beta-weights of this dACC/MCC cluster was negatively associated with the amount of money obtained for oneself compared to that gained for charity (*r*(16) = −0.512, *P* = 0.030), whereas self-focus value orientation was not negatively related to dACC/MCC activity to the same contrast during Stage 2 (even when using small volume correction), and was not associated with the amount of money obtained for self compared to the charity. Lastly, we conducted a mediation analysis probing whether the personal *vs* social activity within the ACC mediates the relationship between the self-focus score and the self *vs* other amount of reward, but this indirect effect was not significant.

No brain region was found to be related to self-focus score during Stage 2. However, four regions (middle temporal gyrus, thalamus, post-central gyrus and precuneus) were engaged during stage 2 in GLM#2 which featured no parametric predictors, see Supplementary Information 6.

The aforementioned findings suggest that dACC/MCC activation is negatively related to the human value orientation (i.e. self-focus value orientation) during foraging (Stage 1), but not during classical economic decision-making (Stage 2), and activity within this cluster is negatively related to the amount of points obtained for oneself compared to charity. This region was not, however, associated with the overall number of forages taken, suggesting that effect of self-focus induced dACC engagement on the overall reward is not underpinned by a general propensity to forage *vs* engage.

#### Discussion

The present study investigated the effect of self-focus on foraging behaviour by examining the role of human value orientations in personal and social foraging behaviour across two experiments. Two main results emerge from this study. First, we demonstrated across two experiments, for the first time, a direct association between one’s human value orientation (in particular one’s degree of self-focus) and the amount of reward earned when foraging for oneself rather than on behalf of a charitable organization. Second, the self-focus score was related to different brain regions depending on the type of decision-making (dACC/MCC during foraging and middle temporal gyrus, thalamus, post-central gyrus and precuneus during classical economic decision-making), and individual variation of dACC/MCC in turn predicted the amount of reward obtained for oneself compared to that given to the charity.

Human values have been previously associated with personal (money obtained for self) and social (money given to a charity) point allocations in classical economic decision-making in the context of charitable donations (Brosch *et al.*, 2011). Specifically, they found that participants who cherished SE values kept more money for themselves instead of donating it to charity. Here, we extend these findings by showing that self-focused value preferences have an effect on behaviour that extends beyond the classical economic decision-making setting to the context of foraging decisions. In particular, across both Experiments 1 and 2, there was a positive association between a self-focus value orientation and the number of points obtained during personal foraging. Moreover, self-focus value orientation was positively associated with the difference in money obtained during personal *vs* social foraging. This is the first demonstration of the effect of human values on point allocation in a task involving foraging computations.

Given the ubiquitous nature of foraging decisions in our daily lives for ourselves and on behalf of others (and the foraging decisions of other on behalf of us), understanding how self-focused *vs* prosocial individuals differently value the welfare of others in a foraging context may have a beneficial societal impact. From a social psychological perspective, the effect of self-focused values on foraging fits the nature of self-focused values. In particular, self-focus has two main value components; (1) OP to Experience and; (2) SE, each of which can be related to foraging behaviour in a unique manner. The first value component, OP to Experience, includes values that challenge the status quo (e.g. ‘creativity,’ ‘curiosity’ and ‘freedom’), while the second value component, SE, includes values that promote personal achievement (e.g. ‘wealth’, ‘success’ and ‘power’). In the case of the binary foraging decision, the option to engage with the currently available option could be conceptualized as accepting the status-quo, while the option to forage can be conceptualized as challenging the status quo. Foraging is inherently about embracing the unknown, with risk but potentially high gains. These gains serve personal achievement even more when the foraging is for the self. It therefore makes sense that individual differences in values can help to explain the substantial individual differences in foraging behaviour.

However, close consideration of specific behavioural parameters in our experiments indicate that neither OP to Experience nor SE orientations behavioural markers alone are sufficient to explain the effects of self-focus. In Experiment 1 there are two status-quo markers: the overall number of forages and the intercept of Stage 1 binary logistic regression, which reflects the overall tendency of the participant towards foraging or engaging. However, there was no significant association between number of forages or the overall tendency to forage and openness values (or self-focus value orientation). In Experiment 2, the variable reflecting status-quo is the harvest option, while the option to travel to a new tree can be conceptualized as challenging the status-quo. The behavioural measures reflecting the extent to which participants are willing to challenge the status-quo are the overall number of times they travel to a new tree, but this variable was not significantly associated with OP to experience. With regard to the role of SE, it could be argued that people with high SE scores are likely to earn more money during personal foraging because they spend more time (i.e. higher reaction time) trying to figure out the optimal choice during the personal *vs* social foraging. However, the reaction time (i.e. personal foraging reaction time minus social foraging reaction time) was not related to the SE (or self-focus) scores in either Experiment 1 or Experiment 2. Overall, then, other mechanisms must be invoked to explain the association between self-focus value orientation and foraging behaviour.

The neuroimaging data from Experiment 1 shed light on this issue. Previous studies have found that dACC is positively associated with the difficulty of a foraging choice (Shenhav *et al.*, 2014; Shenhav *et al.*, 2016b), and that when controlling for choice difficulty previously reported foraging value signals (Kolling *et al.*^2012^) are no longer observed in this region, a finding that was replicated. Previous work found several signals in dACC within foraging-like contexts. In particular, Wittmann *et al.*, (2016) utilized a reward-learning task where participants were asked to engage or forage in a foraging-like patch based on its estimated future value. They found that dACC predicted the positive effects of recent and the negative effects of past rewards on choices. Moreover, they found a graded transition from representation of expected prediction errors to choice moving from posterior to more anterior dACC into the ventromedial prefrontal cortex (vmPFC). Here, we show that dACC activity during foraging is sensitive to an individual’s level of self-focus, even after controlling for task difficulty. Moreover, activation of the dACC during foraging negatively predicted the overall earnings obtained for oneself *vs* the charity. Taken together, our behavioural and imaging findings suggest that more efficient personal foraging and more prominent self-focus was associated with reduced dACC activity.

Beyond these signals that have been observed in dACC within foraging-like contexts the dACC has been implicated in tracking many other signals including potential reward, punishment, errors, surprise effort-reward-trade-off, updating of beliefs, internal models of the environment during learning and the encoding aspects of choice value (the average value of choices afforded by the environment and effort requirements) (Rushworth *et al.*, 2011; Holroyd and Yeung, 2012; Shenhav *et al.*, 2013; Ullsperger *et al.*, 2014; Klein-Flugge *et al.*, 2016; Kolling *et al.*^2016^; Alexander *et al.*, 2017).Taken these findings together, leading researchers to propose a variety of integrative theories proposing that this region combines these varied signals in order to adaptively adjust behaviour, internal models and/or cognitive control states (Holroyd and Yeung, 2012; Rushworth *et al.*, 2012; Shenhav *et al.*, 2013; Kolling *et al.*, 2016; Shenhav *et al.*, 2016a). Generalizing from theories that posit a central role for dACC in the monitoring and/or adjustment of cognitive control, we speculate that self-focused individuals may be less impeded by errors that are likely to occur in their decision-making and thus operate more efficiently in scenarios of high motivational salience (in this case, foraging for personal gain).

One potential limitation of the current study is the final sample size (N = 18) of the imaging experiment compared to the initial sample size (N = 30). The contrast between the self *vs* other conditions did not yield any significant effect differences in the current foraging experiments. Previous work investigating self *vs* other behavioural differences suggested that there may be differences in loss aversion (Mengarelli *et al.*, 2014), in moral decision-making (Crockett *et al.*, 2014), in biases across attention and perception and for charitable decision-making (cited in Sui and Gu, 2017). These prior findings suggest that individuals respond faster when making decisions for themselves regardless of whether they are being selfish, the task is neutral (Lockwood *et al.*, 2015) or even if they are being hyper-altruistic (Crockett *et al.*, 2014). In our design, we did not find differences in reaction time between self and other conditions. One potential contributing factor may be the sample size. However, as discussed in the results section, we fully replicate the main behavioural effects of the original studies. The relatively small sample size may have more impact in the imaging analysis where only 18 participants were included. We appreciate that such a sample size is not optimal for individual differences and mediation analysis. Replication and extension of the current findings need to be investigated in larger sample sizes in future work.

In sum, the present research (a) demonstrated a positive association between the amount of reward obtain during the personal conditions and the human value orientation of self-focus and (b) suggests a novel role for the dACC in influencing foraging behaviour outcomes in social scenarios and paves the way for further studies into psychological and neural differences influencing prosocial and egocentric behaviours.

## Supplementary Material

Supplementary Data
