# Supplementary material for: The effect of self-focus on personal and social foraging behaviour
Source: Soc Cogn Affect Neurosci. 2018 Aug 23;13(9):967–75. doi: 10.1093/scan/nsy057 (PMC6137316; doi:10.1093/scan/nsy057)

Supplementary Information

Supplementary Information 1: Instructions to participants for the foraging Experiment 1 (1.1) and Experiment 2 (1.2).

Before reading the instructions and performing the practise sessions participants were told that they will be playing a game with the goal to maximize the number of points (Experiment 1) or apples (Experiment 2) which will be converted into real money at the end. In addition, participants were apprised that they will be playing these games for themselves for half of the trials and for a charity of their choice for the other half. Participant could select a different charity in Exp2. The list of charities was the following: British Red Cross, Save the Children Fund, The Salvation Army, Oxfam, Cancer Research, and Macmillan Cancer Support.

1.1. "Thank you for participating. Today you will be earning money above pay/credits from coming in. It will be in the form of points shown in a reward bar. These points will turn into cash at the end of the experiment. Please press any key to continue.

You are now going to begin the main task

You will see a blue bar fill up to a golden line as you collect rewards. Each time you fill that bar we will add £0.20 to your final compensation.

Each trial of this task will involve two stages:

In stage 2 you will be choosing between two randomly chosen numbers at a time that ae each GABLES. These numbers represent the amount of reward you get if the gamble wins. These numbers will each have a probability of winning between 20% and 90%. We will show you a purple bar next to each number that represents the probability of getting the reward for that number. The purple bars will perfectly represent the probability of winning for each symbol,

and these probabilities will be assigned at RANDOM before each trial (i.e., they will have nothing to do with the amount of reward you can get from each number).

This all means that if you choose a certain number there is a chance that you will not receive the reward associated it, and that it will not necessarily benefit you to just choose the number associated with higher reward on each trial. On any given trial, it is possible that NEITHER, ONE, or BOTH gambles will be successful, but you will only the reward for the number you chose if it is successful.

Stage 1 takes place before you get to see the probabilities.

During Stage 1 you will simply see the two numbers you could choose between in the middle of the screen. You will ALSO see six other numbers in a box at the top of the screen.

At this stage you can press the A key to choose to swap out the two numbers you were given for two others from that box. When you do this, two numbers will be randomly chosen for that box and the two numbers previously in the middle will go into the box.

You can swap from this box as many times as you'd like. However, there is a cost for each of these swaps. The number of points you would lose is determined by the number to the left of the box.

Whenever you decide you want to stick with the number in the middle of the screen you can press the S key. You will then be shown the probabilities for each number and choose between them.

You will choose between numbers in the middle by pressing the K for the LEFT number and the L key for the RIGHT number. After you make a choice you will see the outcome of BOTH

gambles. A plus (+) will appear next to each gamble that was successful (i.e., the purple bar beat the probability that was chosen), and an X will appear next to each one that was unsuccessful.

If the gamble you chose won, those points will be added to the reward bar. If you were unsuccessful, the bar will stay the same.

Note that each time you choose to swap for a new set of numbers or to make a choice between the numbers in front of you, there will be a few seconds delay before moving on to the next part of the trial. This is normal. You will get a better sense for all of this when you do the practice, but one thing to keep in mind is that the only time you will be able to make a response is when you see question mark (?) in the middle of the screen.

Finally, remember that the only time you can get rewards on this task is during the SECOND stage choice. There are a FIXED number of times total (200) that you will get to make this choice, meaning that you will not get the opportunity for more of these choices if you decide to swap more or fewer times during the first stage. These swaps will only affect which numbers you ultimately choose between, not how many times you will have an opportunity for reward."

1.2. "Thank you for participating! For the next 30 minutes you will be asked to make choices that can earn you money.

In this game, you are in an orchard where you visit trees to harvest apples. You have a limited amount of time in the orchard and you need to decide whether to spend this time harvesting apples at a tree or moving to a brand new tree. Some trees produce more apples than others and each apple is worth money. Your goal is to earn as many apples (money) as possible.

On each trial you will see a tree. You can decide to harvest apples by pressing the Down arrow or move to a new tree by pressing the Right arrow.

Harvesting apples takes up some time but earns you money. However, you will tend to find that the more times you harvest apples at a tree the fewer apples it produces. Each time you harvest, the tree will produce only a fraction of the apples you earned on the previous turn.

Going to a new tree also takes up time but the new tree has never been visited and so has a full supply of apples. There is an endless supply of new trees in each orchard and you will never return to an already visited/harvested tree.

You have a limited amount of time to harvest apples in each orchard and this time starts counting down when you start the game. Harvesting apples takes time and each harvest decision results in fewer apples produced by that tree. Going to a new tree takes time but the tree has not been depleted by apple picking. Your goal is to trade-off time spent harvesting and time spent moving to a new tree.

When the circle at the bottom of the tree turns white you should enter your decision to harvest apples (Down arrow) or move to a new tree (Right arrow). You won't have long to make a choice!

If you don't respond in time, you miss a turn. The more turns you miss, the less time you have left in the game to harvest apples and you will collect less apples (money).

A time out looks like this: “Time Out!” ;

You will harvest apples in different orchards. Some orchards have spread out trees and it takes longer to reach a new tree. Other orchards are more packed together and it takes less time to reach a tree. You will spend a limited and equal amount of time in each type of orchard.

The time it takes to go to a new tree can only change when you switch to a new orchard. Once in an orchard, the time it takes to go to a new tree does not change. You will know when you switch orchards by a change of color, a message and a short break.

You will notice that at some trees you will harvest a larger initial number of apples than at others. The quality of any given tree is random but the likelihood of a good, bad, or average tree never changes. It is unrelated to which orchard you are in or the physical appearance of the tree.

When you harvest apples you may also get portions of whole apples. These are still worth money but at a fraction of a whole apple. You earn the apples on the screen after each harvest. Your goal is to earn as many apples (money) as possible. Your earnings are totaled throughout and paid to you at the end. Every decision counts!

SUMMARY: * Limited and equal time per orchard * Tradeoff time harvesting vs. switching trees * Number of apples produced by each tree varies the same way in every orchard * Harvesting apples depletes the supply in the same way in every orchard * Time to go to a new tree changes only when you switch orchards * Nothing changes until you switch orchards * The apples you earn on every choice count!

Remember, you can earn more by noticing how: * the apples you earn decrease as you harvest from the same tree * the quality of the trees varies * long it takes to go to a new tree or harvest apples.

You are now ready to try a short practice session”

Supplementary Information 2: Parameter values defining the different environment types in Experiment 2

	Long Shallow	Long Steep	Short Shallow	Short Steep
Harvest time (s)	3	3	3	3
Travel time (s)	9	9	6	6
Depletion rate, $\sigma_{depletion\ rate}$	.94, .07	.88, .07	.94, .07	.88, .07
Initial value of a tree (m,σ)	10,1	10,1	10,1	10,1

Supplementary Information 3: Statistical results of GLM#1 and GLM#2 using SPM and SnPM.

GLM#1

	SPM	SnPM
dACC	2 8 38, .011, (624)	2 8 38, .024 (624)

GLM#2

	SPM	SnPM
**Stage 1**		
dACC	-2 6 52, .017, (568)	-2 6 52, .026, (568)
**Stage 2**		
Thalamus	-6 -16 2, .002, (806)	-6 -16 2, .015, (806)
Middle temporal gyrus	-48 -56 10, .023, (464)	-48 -56 10, .034, (464)
Postcentral gyrus	50 -22 50, .002, (843)	50 -22 50, .014, (843)
Precuneus	10 -54 18, .009, (587)	10 -54 18, .021, (587)

Supplementary Information 4: Neuroimaging results of task difficulty collapsing across self and other conditions. Replicating previous findings, dACC activity tracked choice difficulty across the self and other conditions of the two-stage foraging task (only the dACC cluster shown).

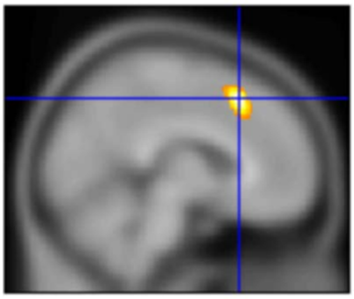

Supplementary Information 5: Bar plots of the raw beta-weights of ACC/MCC seperately during personal, social foraging and their difference. We correlated these beta-weights to the self-focus score. There was a trend of a positive association between self-focus score and the beta-weights during other foraging (r=.451, p=.060), but self-focus score was not associated to the beta-weights during personal foraging (r=.043, p= .865).

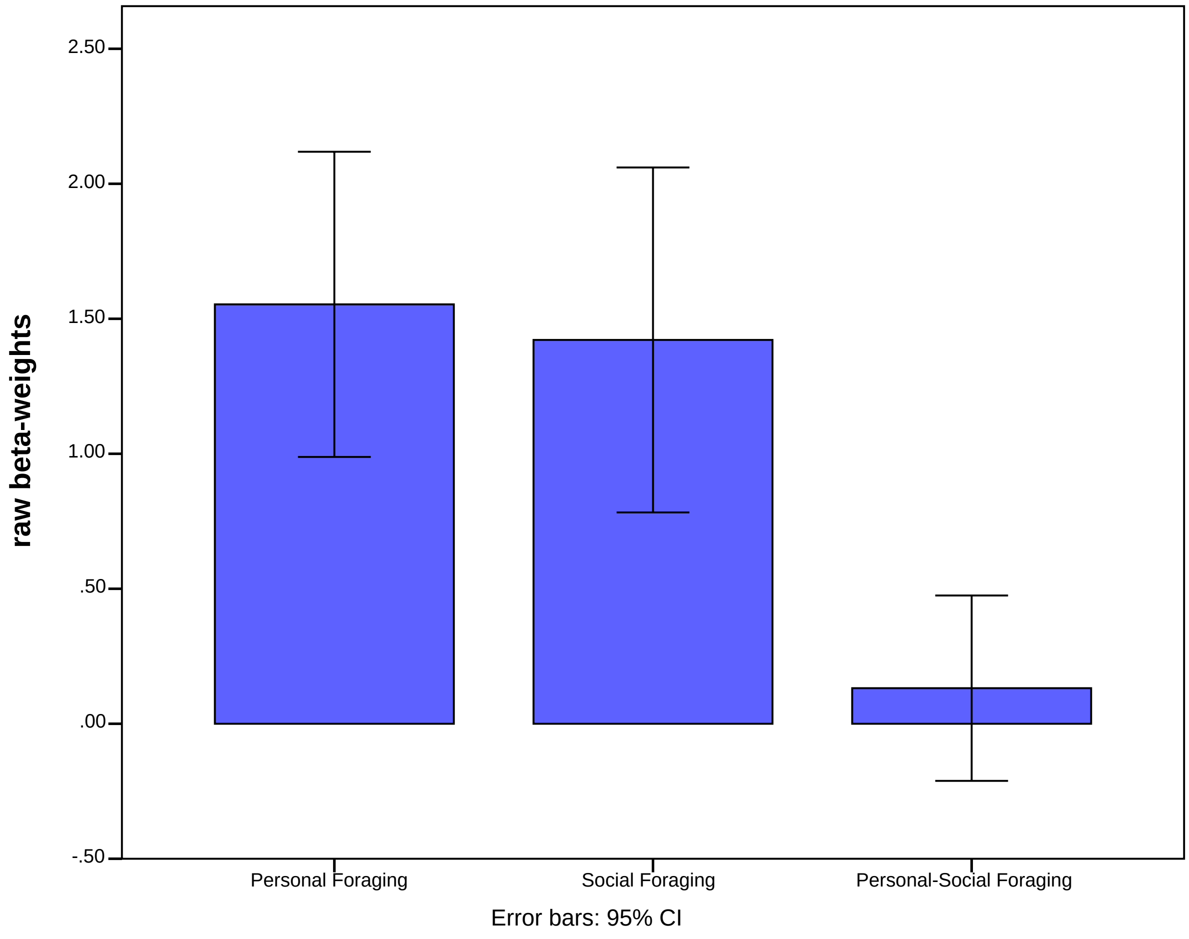

Supplementary Information 6: Neuroimaging results using a general linear model that merely featured the main predictors (stage 1, foraging and stage 2, classical economic decision making).

We probed how self-focus value orientation is associated with the neural activation of the difference between personal and social (i.e., self vs. other) in foraging (Stage 1) and in classical economic decision making (Stage 2). During Stage 1, self-focus was negatively related to activity associated with this contrast in a cluster encompassing dACC

(PFWE-CORR-CLUSTER-level =.0258, x=-2 y=6 z=52 (x=-2 y=18 z=40, x=6 y=10 z=38), k=568).

During Stage 2, self-focus was negatively related to activation in four clusters: (i) thalamus (PFWE-CORR-CLUSTER-level <.015, x=-6 y=-16 z=2, k=806, Figure 6A, see also Table 1), (ii) middle temporal gyrus (PFWE-CORR-CLUSTER-level <.034, x=-48 y=-56 z=10 k=464, Figure 6B), (iii) postcentral gyrus (PFWE-CORR-CLUSTER-level <.014, x=50 y=-22 z=50, k=843, Figure 6C), (iv) precuneus (PFWE-CORR-CLUSTER-level <.021, x=10 y=-54 z=18, k=587, Figure 6D). Similar to the analysis for Stage 1, we investigated whether activation of these regions during Stage 2 was associated with the amount of points obtained for oneself compared to those earned for the charity. Only middle temporal gyrus activation was negatively associated with differential gains, and this association was observed both during Stage 2 (r(16)=-.538, p=.021, two tailed) and during Stage 1 (r(16)=-.559, p=.016, two tailed).

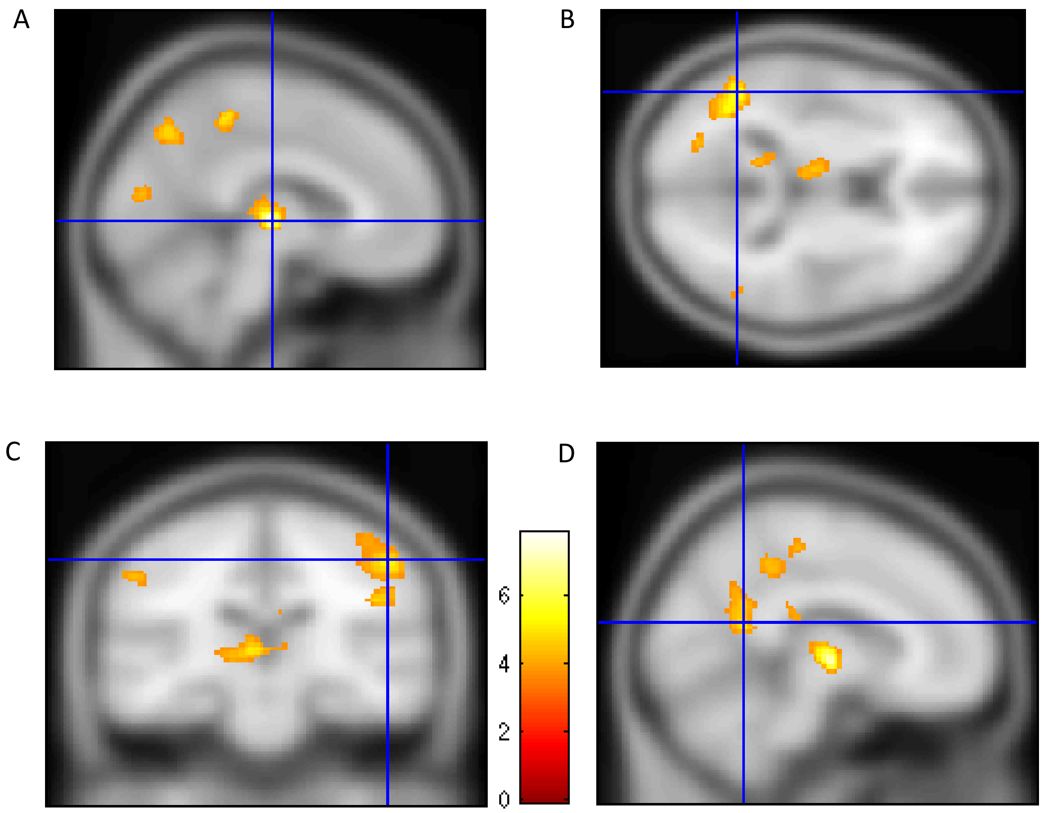

Supplement: Supplementary Data [file nsy057_supp.pdf]
